# Rescuing vascular dysfunction in dorsal pancreatic arteries prevents tacrolimus-induced glucose metabolism disorder in mice

**DOI:** 10.1186/s10020-025-01282-7

**Published:** 2025-06-11

**Authors:** Lingyan Fei, Honghong Wang, Dongliang Zhao, Xiaohua Wang, Jizhen Ren, Lanyun Liu, Chun Tang, Yan Lei, Qingqing Wang, Yuanpeng Nie, Yang Liu, Na Li, Ming Zhong, Nan Xu, Jin Wei, Pontus B. Persson, Andraes Patzak, Pratik H. Khedkar, Zhihua Zheng, Shan Jiang

**Affiliations:** 1 Department of Nephrology, Center of Kidney and Urology, the Seventh Affiliated Hospital, Sun Yat-Sen University, Shenzhen, 518107 China; 2 Department of Physiology, School of Basic Medical Sciences, Zhejiang University School of Medicine, Hangzhou, 310058 China; 3 Department of Pathology, The First Affiliated Hospital of Sun Yat-Sen University, Guangzhou, 510080 China; 4 School of Medicine, The Sun Yat-Sen University, Shenzhen, China; 5 Scientific Research Center, The Seventh Affiliated Hospital of Sun Yat-Sen University, Shenzhen, 518107 China; 6 Department of Physiology and Pathophysiology of School of Basic Medical Sciences and Department of Cardiology of Huaihe Hospital, Henan University, Kaifeng, Henan 475004 PR China; 7 Division of Nephrology at Boston Medical Center, Department of Medicine, Boston University Chobanian and Avedisian School of Medicine, Boston, MA 02118 USA; 8 Institute of Translational Physiology, Charité– Universitätsmedizin Berlin, Corporate Member of Freie Universität Berlin and Humboldt-Universität Zu Berlin, Charitéplatz 1, 10117 Berlin, Germany

**Keywords:** Tacrolimus, Dorsal pancreatic arteries, Vascular dysfunction, Hypoxia, Hyperglycemia

## Abstract

Long-term adverse effects of the immunosuppressant tacrolimus (Tac), such as nephrotoxicity, hepatotoxicity and diabetes, have been widely reported. Up to 33.6% of solid organ transplantation patients receiving Tac treatment develop hyperglycemia; however, the underlying mechanisms remain poorly understood. Here, using a mouse model of Tac-induced hyperglycemia, we found that Tac-induced body-weight loss, hyperglycemia, hypoinsulinemia, glucose intolerance and insulin resistance were improved by valsartan, a renin-angiotensin system (RAS) inhibitor. Histological and immunofluorescence analysis of the pancreas showed reduced islet areas and β-cell mass in Tac-treated mice. Moreover, when compared to control mice, isolated islets from Tac-treated mice showed a downregulation of cell-proliferation markers (Ki67, Ccna2 and Ccnd1) while an upregulation of apoptotic markers (DNA fragmentation, Bax and Caspase3). Tac also upregulated hypoxia-related markers in the pancreas, including hypoxia-inducible factor-1α (HIF-1α) and its downstream factors (Adm, Hmox1 and Vegfa), CD31 and pimonidazole adducts. Furthermore, treatment with Tac led to vascular dysfunction in pancreatic arteries. All of these adverse effects could be partially or fully abrogated by valsartan. Tac also increased levels of renin in renal tissue (1.00 ± 0.06 vs 1.29 ± 0.04, *p* < 0.05) and serum (28.35 ± 4.29 ng/mL vs 51.99 ± 4.95 ng/mL, *p* < 0.05). Inhibition of RAS by valsartan protected against Tac-induced vascular dysfunction in renal interlobar arteries. Collectively, our data illustrate a previously undescribed mechanism, in which Tac-induced vascular dysfunction in renal interlobar arteries leads to RAS activation. Blocking RAS by valsartan alleviates vascular dysfunction in dorsal pancreatic arteries and hypoxia in islets, which in turn prevents Tac-induced β-cell dysfunction and glucose metabolism disorder.

**Supplementary Information:**

The online version contains supplementary material available at 10.1186/s10020-025-01282-7.

## Introduction

For patients with irreversible end-stage organ failure, solid organ transplantation (SOT) is the optimal therapeutic option (Jenssen and Hartmann 2019; Larsen et al. 2016). However, transplant patients are at a high risk of cardiovascular disease, diabetes mellitus and premature death, probably owing to the prolonged usage of immunosuppressive drugs (Jenssen and Hartmann 2019, 2015). About 10–40% of patients receiving SOT end up developing post-transplant diabetes mellitus (PTDM) during the first year (Jenssen and Hartmann 2019, 2015). While PTDM shares common risk factors with type 2 diabetes mellitus (T2DM), such as central obesity, age, inflammation, genetic susceptibility, PTDM-specific risk factors including the use of glucocorticoids, calcineurin inhibitors (CNIs) and mTOR inhibitors also play a role (Jenssen and Hartmann 2019). PTDM itself is an independent risk factor for graft failure (Lin et al. 2021), premature cardiovascular disease (Pilmore et al. 2010) and mortality (Larsen et al. 2016; Lin et al. 2021; Pham et al. 2024; Cosio et al. 2002). Nevertheless, the precise mechanisms leading to PTDM remains elusive. Therefore, there is an urgent need to uncover new mechanisms for PTDM so that new therapeutic targets can be identified for future treatment.

Tacrolimus (Tac) has been and remains a widely prescribed immunosuppressive drug for preventing allograft rejection after organ transplantation and treating autoimmune diseases (Hoorn et al. 2011; Kaufman et al. 2021; Tacrolimus 2020; Ong and Gaston 2021). At the same time, prolonged use of Tac can cause numerous adverse effects, including hypertension (Zhou et al. 2022; Wei et al. 2024), hyperkalemia (Duan et al. 2024), nephrotoxicity (Ko et al. 2022; Zmijewska et al. 2021) and PTDM (Jiao et al. 2020a; Rodriguez-Rodriguez et al. 2019). While the mechanisms underlying Tac-induced PTDM are not fully understood, the key features of its pathogenesis include β-cell dysfunction, alterations in the gut microbiota (Jiao et al. 2020a; Li et al. 2024), inhibition of glucose uptake in adipocytes (Wyzgał et al. 2007), and impaired transcription of insulin genes (Jenssen and Hartmann 2019; Hernández-Fisac et al. 2007). Multiple studies highlight the importance of islet blood flow in regulating insulin secretion (Hashimoto et al. 2015; Jansson et al. 2016; Richards et al. 2010; Islam et al. 2024). Islet blood flow is closely linked to pancreatic blood flow, which is mediated by dorsal pancreatic arteries (Jansson et al. 2016; Muratore et al. 2021). There is increasing evidence suggesting that modulation of the vascular tone may contribute to glucose intolerance and insulin resistance (Islam et al. 2024; Obata et al. 2019).Considering that prolonged exposure to Tac causes vascular dysfunction (Ping et al. 2017; Wang et al. 2022; Chiasson et al. 2011), we hypothesized that Tac-induced vascular dysfunction of dorsal pancreatic arteries results in pancreatic hypoperfusion thereby impairing islet endocrine function. To test this hypothesis, we first established a mouse model of Tac-induced hyperglycemia. Using qPCR and immunohistochemistry, we investigated the molecular mechanisms underlying Tac-induced diabetes. Vascular function of dorsal pancreatic and renal interlobar artery was assessed by wire myography. Ultimately, we uncovered that vascular dysfunction in the dorsal pancreatic arteries plays a key role in the development of Tac-induced glucose metabolism disorder.

## Methods

### Experimental animal

Male C57Bl/6 mice (8–10 weeks old) were obtained from SLAC laboratory animal company (Shanghai, China). Mice were housed in a temperature-controlled facility with a 12 h dark–light cycle and were allowed free access to tap water and a standard diet. They were randomly divided into four groups and allowed to acclimatize for two weeks. Next, one group of mice received valsartan (Val, 30 mg/kg/day (Liu et al. 2021; Suematsu et al. 2016), S1-3, V129241, Aladdin, China) by gavage. The second group received Tac (5 mg/kg/day (Pietropaolo et al. 2015; Xie et al. 2022; Tourret et al. 2017), HY-13756, MCE, USA) intraperitonially. The third group received both Tac and Val, while the fourth group received solvent only and served as control. All groups were treated for 6 months during which the body weight was recorded every two weeks.

### Blood glucose, plasma insulin measurements and HOMA-IR, HOMA-β index calculation

Random and fasting blood glucose were measured by an Accu-Check glucose meter. Plasma insulin levels were assessed by ELISA kit (CSB-E05071 m, Cusabio, China) according to the manufacturers’ instructions. Homeostatic Model Assessment of Insulin Resistance (HOMA-IR) and β-cell function (HOMA-β) were calculated as follows: HOMA-IR = fasting plasma insulin (mIU/L) × fasting blood glucose (mmol/L)/22.5 and HOMA-β = 20 × fasting plasma insulin (mIU/L)/[fasting blood glucose (mmol/L)−3.5].

### GTT and ITT measurements

For glucose tolerance test (GTT), four groups of mice were fasted for 16 h before D-(+)-glucose (1.5 g/kg) was injected intraperitoneally. For insulin tolerance test (ITT), four groups of mice were fasted for 6 h before insulin (1 U/kg body weight) was administered intraperitoneally. In both cases, blood glucose levels were measured once before the injection (baseline fasting blood glucose) and at 30, 60, 90 and 120 min after the injection.

### Blood flow measurements

To quantify pancreas and renal blood flow, we utilized the non-radioactive black microspheres technique as described previously (Lai et al. 2007; Jansson at al. 1983). Briefly, mice were anaesthetized with isoflurane (2%) and positioned on a temperature-controlled table, which was used to maintain their body temperature at 37.0 ± 1.0 °C. Then, the left carotid artery and left femoral artery were isolated and cannulated using a polyethylene catheter (PE-10) for microsphere injection and arterial blood sample collection, respectively. After a 10-min resting period, approximately 1.5 × 10^–5^ non-radioactive black microspheres (diameter 10 µm, 100 µl, E–Z Trac, IMT; Irvine, USA) were injected through the carotid artery into the ascending aorta. After injection, arterial blood was collected using the catheter placed in the femoral artery and a syringe pump at a constant rate of 0.4 ml/min for 60 s. Then, the pancreas and kidney were removed and weighed. A freeze–thawing technique was used to visualize the microspheres in the organs. The number of microspheres in the pancreas and kidney was then counted under a microscope. The reference blood sample was dissolved in 16 mol/l KOH + 2% (vol./vol.) Tween 80 and the number of microspheres was counted. Pancreatic and renal blood flows were calculated using the formula, Q_org_ = N_org_ x Q_ref_/N_ref_, where Q_org_ is the organ (pancreas or kidney) blood flow (ml/min), Q_ref_ is the rate of the blood sample collection (ml/min), N_org_ and N_ref_ are the number of microspheres in the organ and reference blood sample, respectively.

### Measurement of vasoreactivity in dorsal pancreatic and renal interlobar arteries

Vascular contraction and dilation in resistance arteries were assessed by wire myography, as we previously described (Wang et al. 2022; Jiang et al. 2021; Hu et al. 2021; Fei et al. 2023). In brief, mice from the control, Val, Tac and Tac + Val groups were anesthetized under 2% isoflurane and then sacrificed. The dorsal pancreatic and renal interlobar arteries were dissected under a stereomicroscope. Next, the dorsal pancreatic and renal interlobar arteries were isolated and placed in ice-cold Krebs–Henseleit solution, containing 112 mM NaCl, 5 mM KCl, 25 mM NaHCO_3_, 1 mM NaH_2_PO_4_, 0.5 mM MgCl_2_, 2.5 mM CaCl_2_ and 11.5 mM glucose. The arteries were cut into rings (2 mm in length). mounted onto the wire myograph system and allowed to warm up to 37 °C for 10 min. The resting tension was set according to manufacturer’s protocol. Following this, vessel reactivity was ascertained by applying a high-potassium solution (KPSS, Krebs-Hanseleit solution with 80 mM KCl) followed by a wash with Krebs-Hanseleit solution. Finally, cumulative dose response curves to angiotensin II (Ang II, 10^–12^ to 10^–6^ mol/L), U46619 (10^–6^ to 10^–11^ mol/L), phenylephrine (PE, 10^–10^ to 10^–5^ mol/L) and ACh (10^–9^ to 10^–5^ mol/L) were obtained.

### Histologic, immunohistochemical, and immunofluorescence analyses

Pancreases were isolated from all four groups of mice (control, Val, Tac and Tac + Val) and fixed using 4% paraformaldehyde at 4 °C overnight. The fixed pancreatic tissue samples were embedded with paraffin and sliced at 4 μm thickness. Next, sections were stained using the hematoxylin & eosin (H&E), immunofluorescence or immunohistochemical methods. Sections stained using immunofluorescence and immunohistochemistry were used to estimate gene expression semi-quantitatively as percentage of positively stained cells in 10 random fields per section.

### Plasma renin and ang II measurements

Plasma Ang II and renin levels were measured by the Mouse Ang II ELISA Kit (E-EL-M2612, Elabscience Biotechnology, China) and the Mouse REN (Renin) ELISA Kit (E-EL-M0061, Elabscience Biotechnology, China), respectively, according to the manufacturer’s instructions.

### Pancreatic islet isolation

Islets from the pancreas were isolated from all four groups (control, Val, Tac and Tac + Val) as previously described (Obata et al. 2019). In brief, the entrance to the duodenum was ligated. The bile duct was injected with 2.5 ml of 0.24 g/l Liberase TL (05401020001, Roche Diagnostics, Japan) prepared in Hanks’ Balanced Salt Solution (HBSS) containing 25 mmol/l HEPES. The expanded pancreas was removed and digested at 37 °C for 24 min. The digested pancreatic tissue was dispersed by gentle pipetting and washed twice with ice-cold HBSS containing 25 mmol/l HEPES and 10% FBS. Islets were hand-picked under a stereoscopic microscope and used immediately for experiments.

### Quantitative Polymerase Chain Reaction (qPCR)

Total RNA in isolated islets was extracted using an RNeasy mini kit (Qiagen, Valencia, USA) according to the manufacturer’s instructions. mRNA was then reverse transcribed into cDNA using a commercial kit (Bio-rad, USA). qPCR was then performed by using SYBR Green supermix (Bio-rad, USA). All primers are listed in Supplementary Table S1. The comparative Ct method (2^−ΔΔCt^) was used to calculate the relative mRNA expression level.

### Statistical analyses

Statistical analyses were performed with Prism 10. Data represent means ± SEM from at least three independent experiments. Statistical differences were analyzed by one-way ANOVA followed by Tukey’s post hoc test. Differences with *P* < 0.05 were considered statistically significant and all *P* values are two-sided.

## Results

### RASi relieves Tac-induced glucose metabolism disorder

**Fig. 1:**
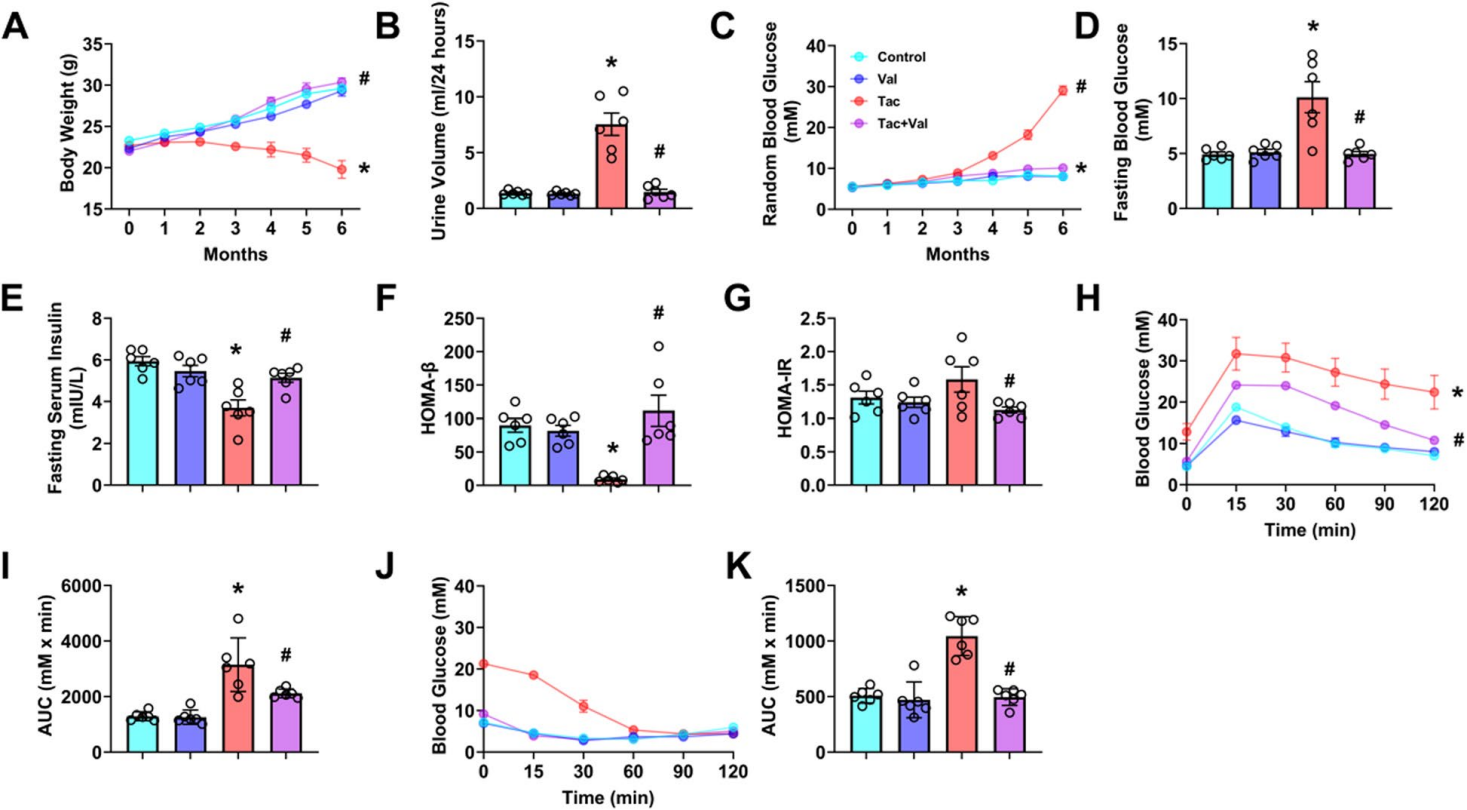
Inhibition of RAS protected mice against Tac-induced glucose metabolism disorder. **A** Time course of body weight change in mice treated with vehicle, Tac and/or Val. **B** 24-h urine output in vehicle (control), Val, Tac and Tac + Val treated mice. **C** Time course of random blood glucose change in mice treated with vehicle, Tac and/or Val. The levels of (**D**) fasting blood glucose and (**E**) fasting serum insulin in control, Val, Tac and Tac + Val mice. **F** HOMA-β index, (**G**) HOMA-IR index, (**H**) GTT, (**I**) area under the curve (AUC) of GTT, (**J**) ITT and (**K**) AUC of ITT in four groups of mice. (*p < 0.05 versus Control, #*p* < 0.05 versus Tac). Statistical differences were calculated by two-way ANOVA followed by Dunnett’s post hoc test (**A**, **B**, **H** and **J**), one-way ANOVA followed by Turkey’s post hoc test (**C**-**G**, **I** and **K**)

We first investigated whether administration of the RAS inhibitor (RASi) Val could alleviate Tac-induced glucose metabolism disorder. To this end, Tac and/or Val were administered to C57bl/6 mice for 6 months. Mice treated with Tac alone suffered a sustained weight loss (Fig. 1A), polyuria (Fig. 1B) and had elevated blood glucose levels in both random (Fig. 1C) and fasting (Fig. 1D) samples. Moreover, Tac-treatment also caused significantly lower fasting serum insulin levels (Fig. 1E) and higher serum creatinine (Figure S4 A) and blood urea nitrogen (BUN, Figure S4 B). While, serum K^+^ was significantly lower (Figure S5) in Tac-treated mice, serum Mg^2+^ (Figure S6) was slightly higher, but not significantly. At the same time, the values of homeostatic model assessment of β-cell function (HOMA-β, Fig. 1F) were significantly lower while homeostatic model assessment of insulin resistance (HOMA-IR, Fig. 1G) were slightly higher, however not significantly, in Tac-treated mice. As expected, Tac-treated mice exhibited higher glucose levels during intraperitoneal GTT (Fig. 1H) as well as a significantly higher area under the curve (AUC, Fig. 1I) of the GTT. Likewise, blood glucose levels (Fig. 1J) and the AUC (Fig. 1K) of ITT were also significantly higher. All of these effects of Tac were abolished in mice co-treated with Val.

### RASi mitigates Tac-induced β-cell dysfunction

**Fig. 2:**
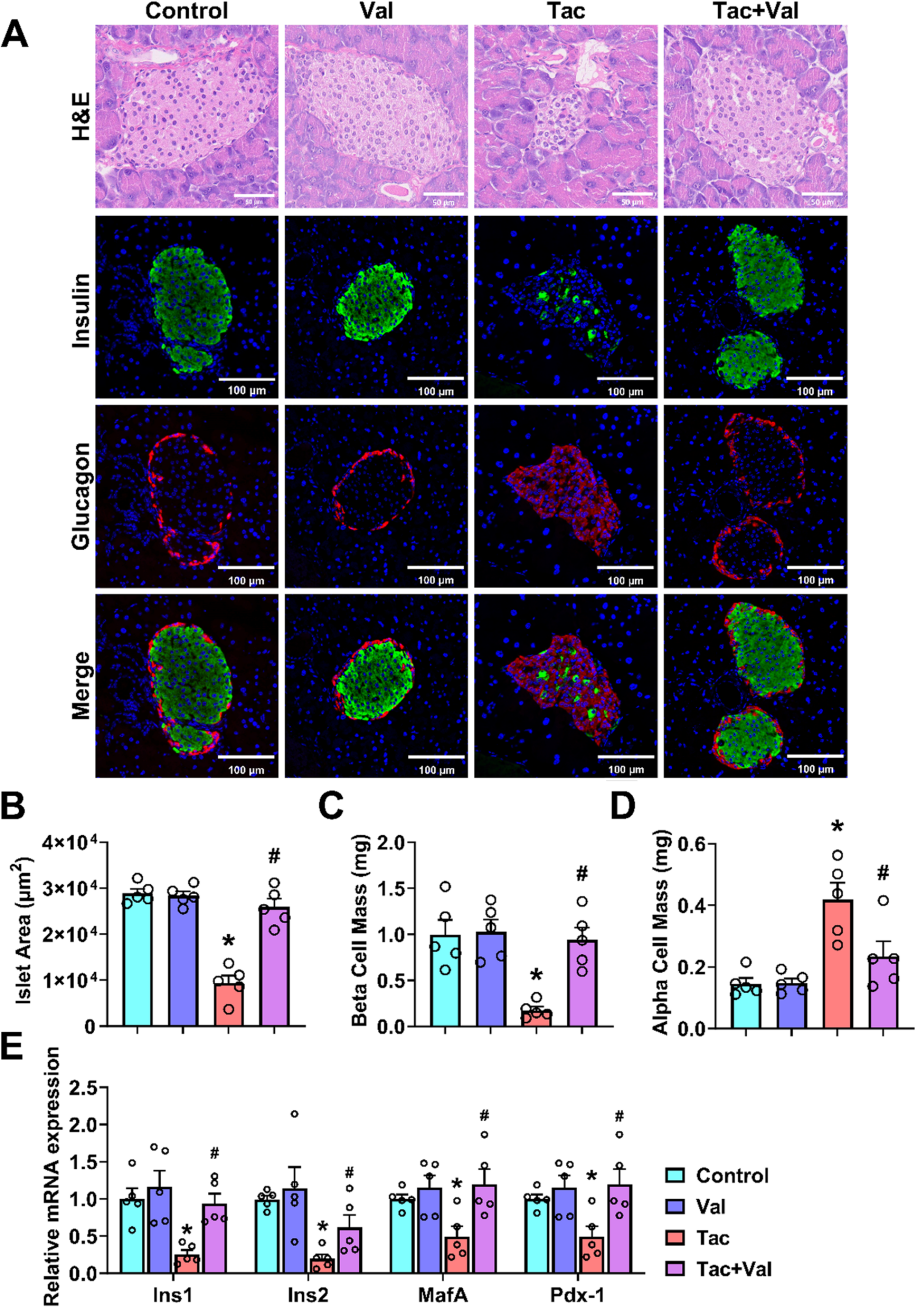
Inhibition of RAS protected against β-cell dysfunction induced by Tac. **A** Representative photomicrographs of islets stained with H&E, insulin (green) and glucagon (red) from mice treated with vehicle, Tac and/or Val. **B** The pancreatic islet areas from the four groups were measured (*n* = 5). Quantification of beta (**C**) and alpha (**D**) cell mass (*n* = 5). **E** The mRNA levels of insulin biosynthesis-related genes (*n* = 3). (*p < 0.05 versus Control, #*p* < 0.05 versus Tac). Statistical differences were calculated by one-way ANOVA followed by Turkey’s post hoc test (**B**-**E**)

As outlined above, Tac-induced glucose metabolism disorder was abrogated by treatment with RASi. Since β-cell function plays a central role in mediating glucose metabolism, we investigated the effect of Tac and RASi on pancreatic islets histologically (Fig. 2A). Tac-treated mice indeed displayed a significantly reduced pancreatic islet area compared to control (Fig. 2B). Insulin and glucagon double staining revealed that Tac treatment caused a significant reduction in the β-cell mass (Fig. 2C) while the α-cell mass had increased (Fig. 2D). mRNA expression of genes related to insulin biosynthesis, including Ins1, Ins2, MafA and Pdx-1, was downregulated in Tac-treated mice (Fig. 2E). All of these effects were mitigated by co-treatment with RASi. These data suggest that RASi abrogates Tac-induced glucose metabolism disorder by preserving β-cell function.

### RASi improves Tac-induced imbalance of β-cell proliferation and apoptosis

**Fig. 3:**
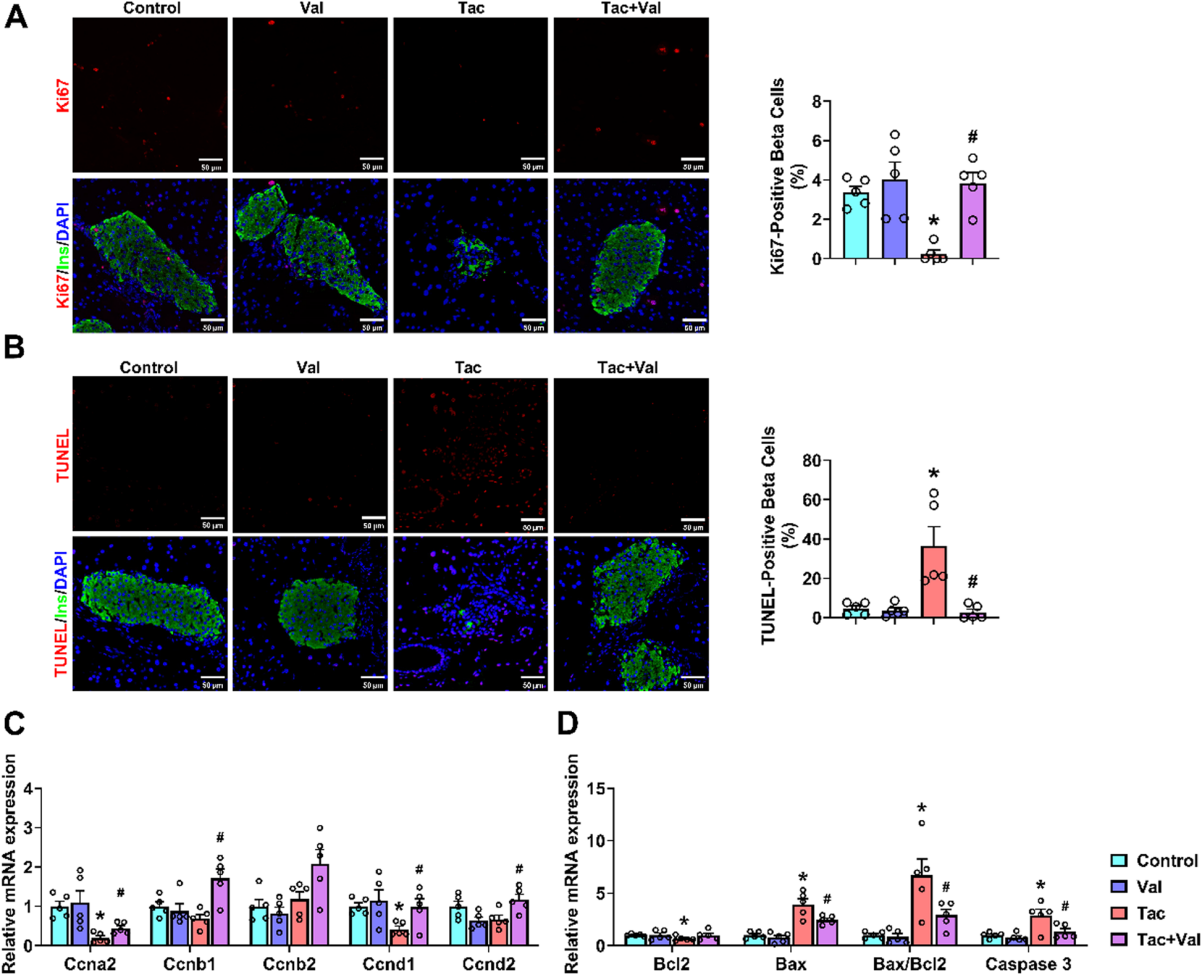
Inhibition of RAS improves the abnormal proliferation and apoptosis of β-cell induced by Tac. **A** Representative photomicrographs, and quantification of Ki67 positive β-cell (*N* = 5). **B** Representative photomicrographs, and quantification of TUNEL positive β-cell (*N* = 5). The mRNA levels of cell proliferation (**C**) and apoptosis (**D**) 867Related genes (*n* = 3). (*p < 0.05 versus Control, #*p* < 0.05 versus Tac). Statistical differences were calculated by one-way ANOVA followed by Turkey’s post hoc test (**A**-**D**)

Since the imbalance between proliferation and apoptosis is a major underlying cause of β-cell dysfunction (Liu and Li 2021; Hasib et al. 2017; Wajchenberg 2007; Thomas et al. 2021), we investigated if Tac and RASi either alone or in combination affect these processes. To this end, we costained pancreatic sections for insulin and Ki67 or TUNEL. Interestingly, Tac treatment downregulated the β-cell proliferation marker Ki67 (Fig. 3A) but increased β-cell apoptosis (Fig. 3B), both of which could be reversed by co-treatment with RASi. Tac also reduced the mRNA levels of the proliferation markers Ccna2 and Ccnd1, an effect that could likewise be reversed by RASi co-treatment (Fig. 3C). Similarly, RASi could also reverse the Tac-induced upregulation of the pro-apoptotic Bax and Caspase 3 genes, and downregulation of the anti-apoptotic gene Bcl2 (Fig. 3D). We conclude from these data that RASi improves Tac-induced β-cell dysfunction by reducing the imbalance between proliferation and apoptosis.

### RASi alleviates Tac-induced pancreas hypoxia in the pancreas

**Fig. 4:**
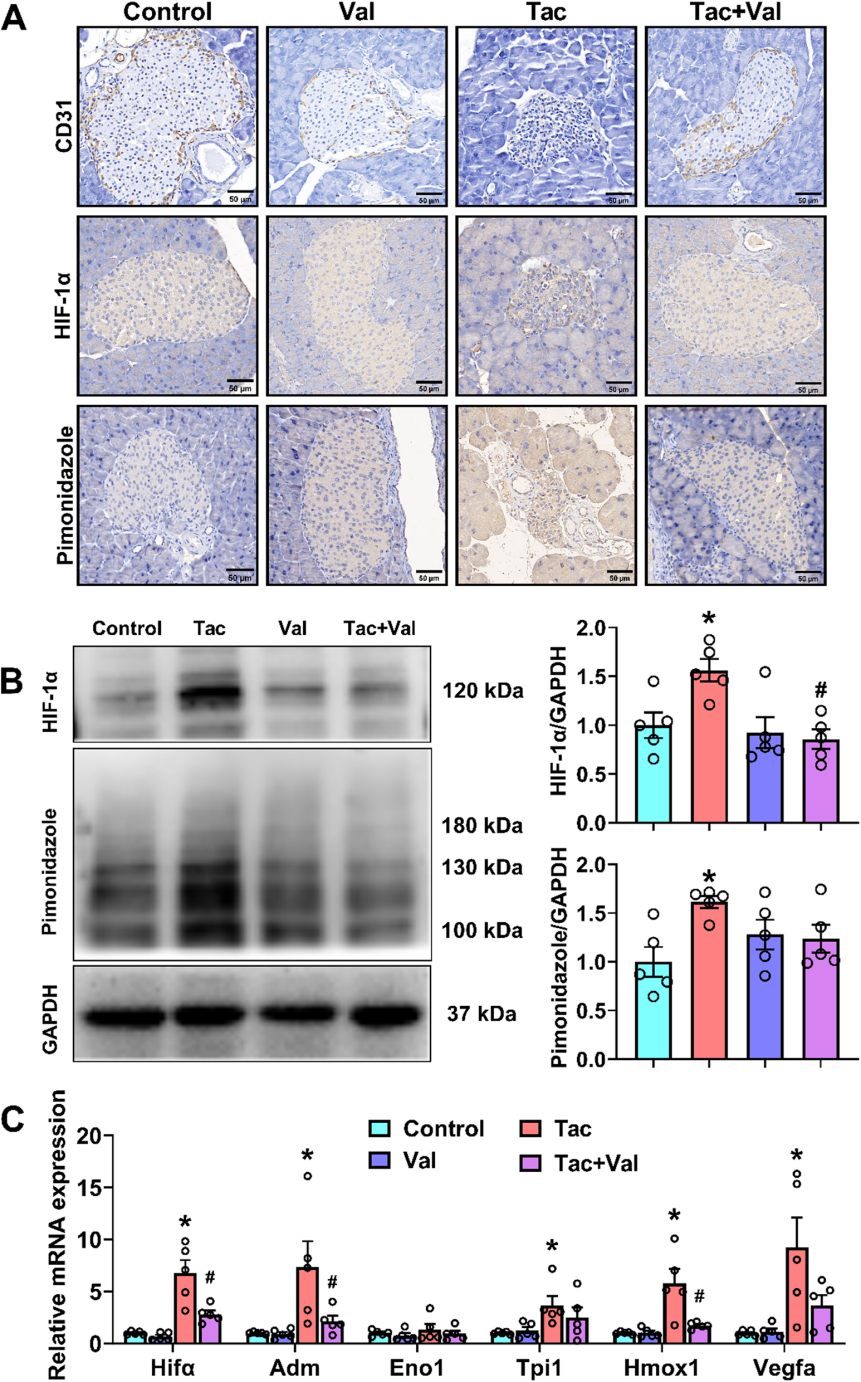
Inhibition of RAS protected against islet hypoxia induced by Tac. **A** CD31, HIF-1α and pimonidazole immunostaining of pancreas sections (*N* = 5). **B** Representative immunoblotting and quantification of HIF-1α and pimonidazole adducts in islets from the four groups (*N* = 4). **C** The mRNA levels of HIF-1α and its downstream target genes (*n* = 3). (*p < 0.05 versus Control, #*p* < 0.05 versus Tac). Statistical differences were calculated by one-way ANOVA followed by Turkey’s post hoc test (**A** and **C**)

It is now well accepted that hypoxic stress plays an important role in β-cell dysfunction (Gunton 2020; Ilegems et al. 2022). However, it is unclear whether hypoxic stress is also involved in Tac-induced β-cell dysfunction. Isolated islets from Tac-treated mice showed decreased CD31-positive areas and increased pimonidazole-positive areas (Fig. 4A). CD31 is a marker for blood vessels while pimonidazole adducts indicate a hypoxic state. Thus, the islets from the Tac-treated mice were in a stronger hypoxic state and had less capillary density compared to control mice. Another marker for hypoxia, viz. hypoxia-inducible factor 1α (HIF-1α), was also upregulated under Tac treatment as assessed by immunohistochemistry (Fig. 4A), Western blot (Fig. 4B) and qPCR (Fig. 4C). Moreover, downstream genes of HIF-1α, such as Adm, Hmox1 and Vegfa, were upregulated in Tac-treated mice. All of them could be partially or fully rescued by co-administration of RASi (Fig. 4C). Together, these data highlight the important role of pancreatic hypoxia in Tac-induced glucose metabolism disorder.

### RASi rescues Tac-induced vascular dysfunction of dorsal pancreatic artery

**Fig. 5:**
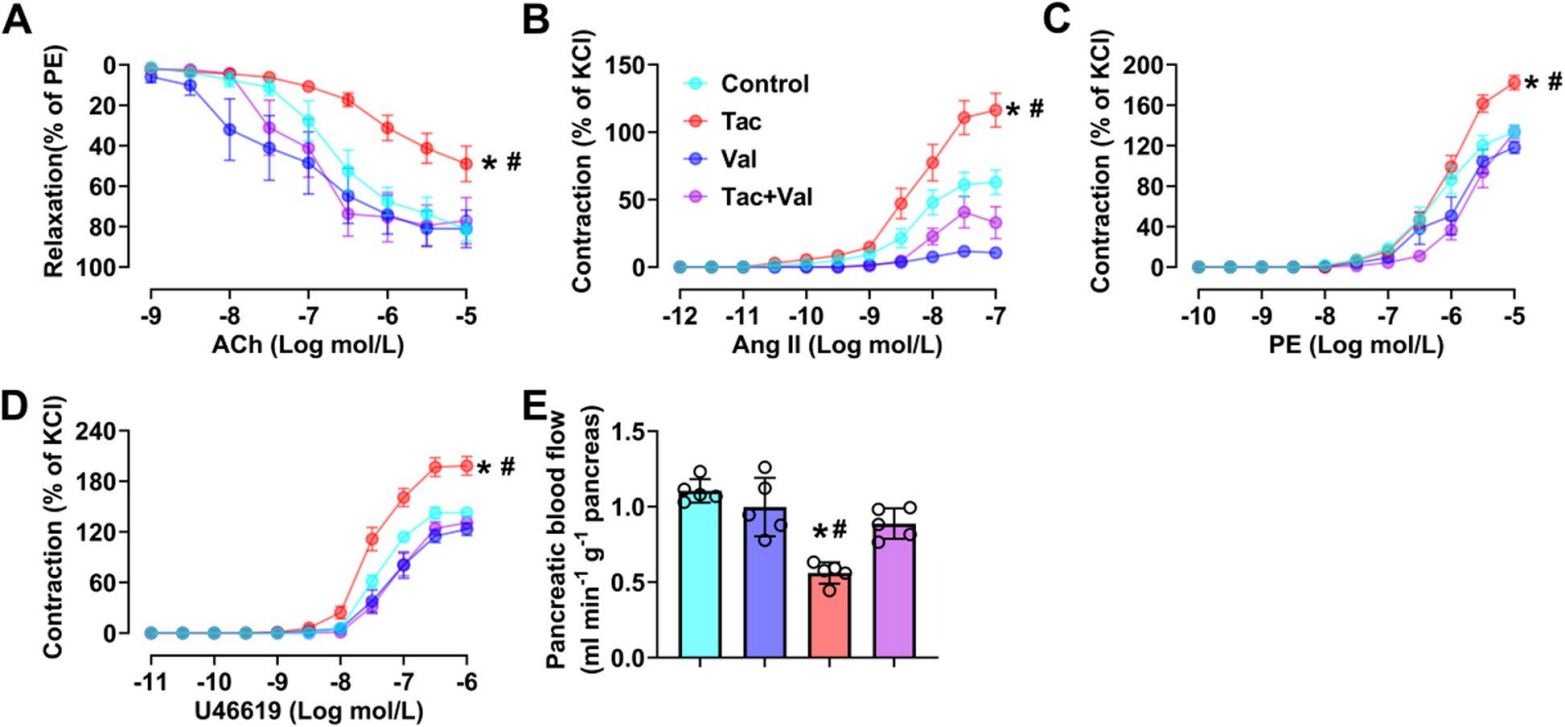
Inhibition of RAS protected against vascular dysfunction induced by Tac in dorsal pancreatic arteries. **A** ACh-induced vasorelaxation in dorsal pancreatic arteries from mice treated with vehicle, Tac and/or Val (*N* = 6). **B** Ang II, (**C**) PE and (**D**) U46619-induced vasoconstriction in dorsal pancreatic arteries from mice treated with vehicle, Tac and/or Val (*N* = 6). **E** The change in pancreatic blood flow in mice treated with vehicle, Tac and/or Val (*N* = 6). (*p < 0.05 versus Control, #*p* < 0.05 versus Tac). Statistical differences were calculated by two-way ANOVA followed by Dunnett’s post hoc test (**A**-**D**). Statistical differences were calculated by one-way ANOVA followed by Turkey’s post hoc test (**E**)

The pancreatic blood flow plays a critical role in regulating insulin secretion by mediating oxygen supply to islets (Nyström et al. 2012; Huang et al. 2007). It is mainly controlled by splenic artery and dorsal pancreatic artery (Muratore et al. 2021). Therefore, we next examined the change in pancreatic blood flow and vascular tone of dorsal pancreatic artery. As assessed by wire myography, ACh-induced vasorelaxation of the dorsal pancreatic artery was impaired in Tac-treated mice (Fig. 5A), while vasoconstriction to angiotensin II (Ang II), phenylephrine (PE) and U46619 was enhanced (Fig. 5B-D). A significantly reduced pancreatic blood flow was observed when mice were administered with Tac (Fig. 5E). Co-administration of RASi could markedly reduce Tac-induced vascular dysfunction of dorsal pancreatic arteries and hypoperfusion of the pancreas.

### Renin-angiotensin system (RAS) is hyperactivated in Tac-treated mice

**Fig. 6:**
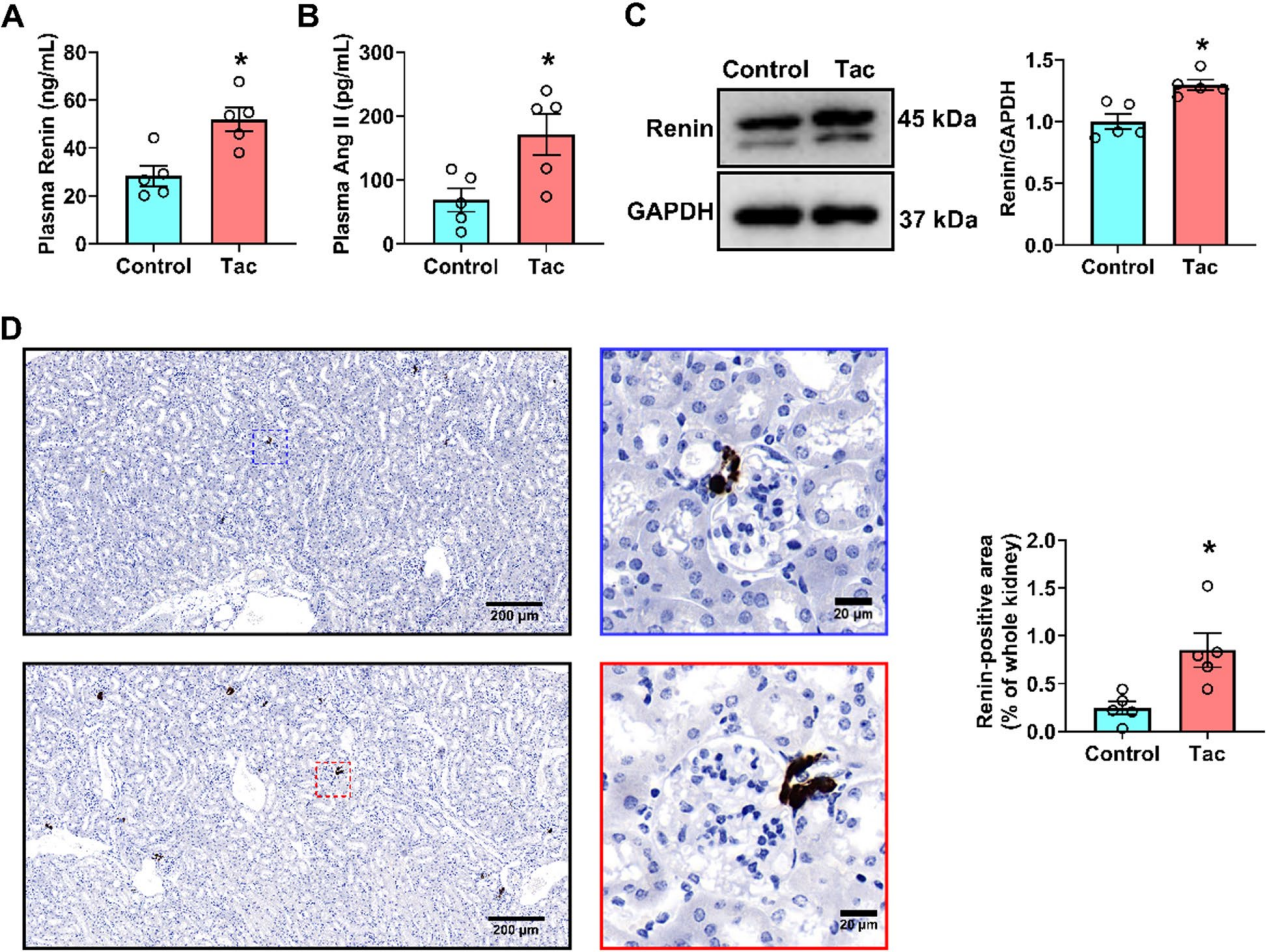
Administration of Tac activate systemic RAS. The changes in plasma renin (**A**) and Ang II (**B**) levels in mice treated with vehicle or Tac (*N* = 5). **C** The protein levels of renin in kidney were detected by western blot (*N* = 4). **D** Representative photomicrographs, and quantification of renin expression in kidney sections (*N* = 5). (*p < 0.05 versus Control). Statistical differences were calculated by the paired T-test

To further investigate the role of RAS in Tac-induced glucose metabolism disorder, we measured the levels of renin and Ang II in serum and renal tissue. Serum Ang II (Fig. 6A) and renin levels (Fig. 6B) were enhanced under Tac treatment. Renin expression was also markedly elevated in the renal tissue of Tac-treated mice as revealed by IHC staining (Fig. 6D) and western blot (Fig. 6C). These results indicate that Tac treatment activated the RAS.

### RASi alleviates Tac-induced renal hypoperfusion and vascular dysfunction

**Fig. 7:**
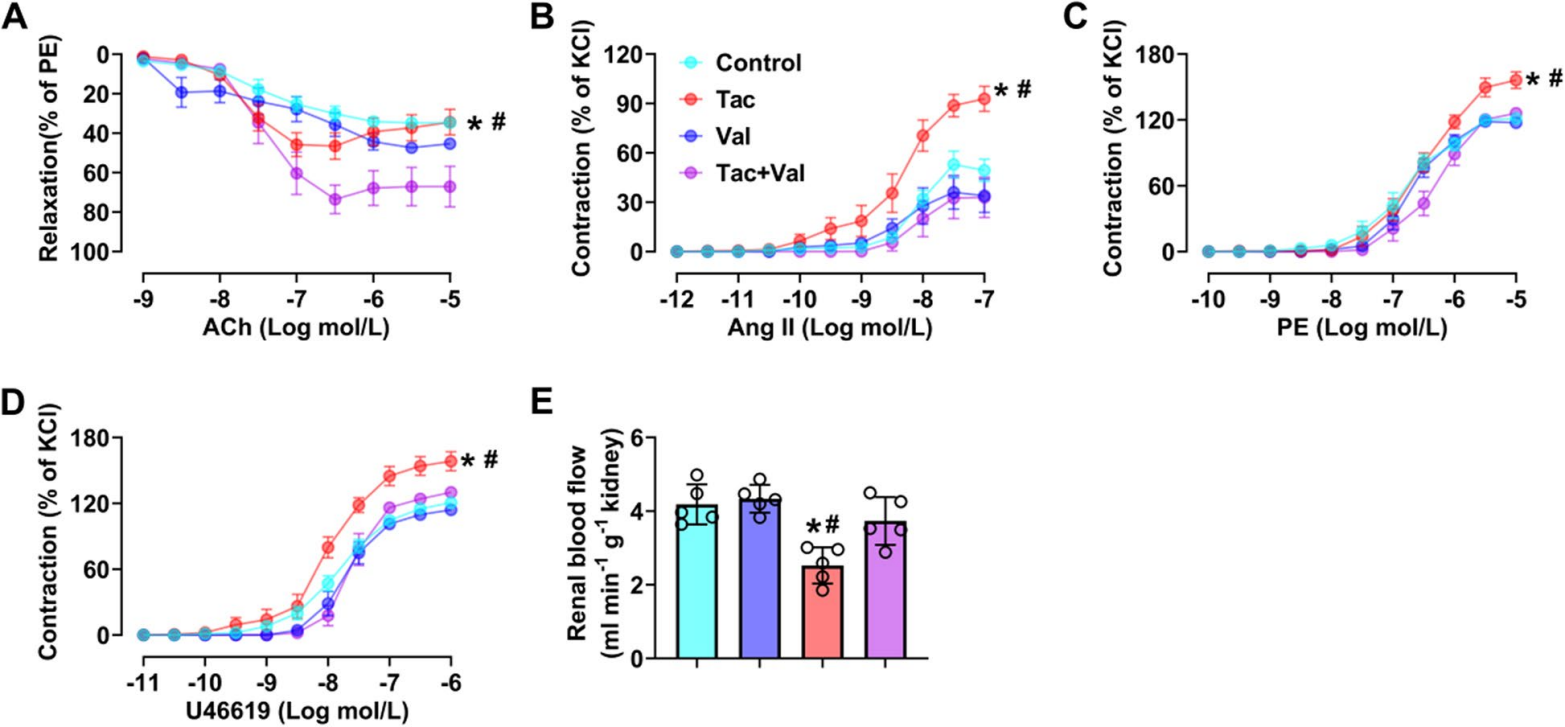
Inhibition of RAS protected against vascular dysfunction in renal arteries induced by Tac. **A** ACh-induced vasorelaxation in renal interlobar arteries from mice treated with vehicle, Tac and/or Val (*N* = 6). (**B**) Ang II, (**C**) PE and (**D**) U46619-induced vasoconstriction in renal interlobar arteries from mice treated with vehicle, Tac and/or Val (*N* = 6). **E** The change in renal blood flow in mice treated with vehicle, Tac and/or Val (*N* = 6). (*p < 0.05 versus Control, #*p* < 0.05 versus Tac). Statistical differences were calculated by two-way ANOVA followed by Dunnett’s post hoc test (**A**-**D**). Statistical differences were calculated by one-way ANOVA followed by Turkey’s post hoc test (**E**)

Dysfunction of renal arteries leads to renal hypoperfusion and hypoxia. The latter in a chronic situation is known to increase renin as well as Ang II levels in the plasma (Gomez and Sequeira-Lopez 2018; Kong et al. 2023). Therefore, we hypothesized that Tac-induced activation of RAS is a result of dysfunction in renal arteries and the consequent renal hypoperfusion and hypoxia. As assessed by wire myography, renal arteries from Tac-treated mice did not only show impaired vasorelaxation to ACh (Fig. 7A) but also stronger contraction to Ang II, PE, as well as U46619 (Fig. 7B-D). Moreover, these effects could be partially or fully reversed by co-administration of Val. These results substantiate the notion that Tac-induced RAS activation is caused by renal hypoperfusion and hypoxia, which are in turn caused by vascular dysfunction in renal arteries.

## Discussion

Although a large body of evidence confirms the direct cytotoxic effects of Tac on β-cells (Triñanes et al. 2017; Ko et al. 2022; Park et al. 2023), whether vascular dysfunction is involved in Tac-induced glucose metabolism disorder remains unclear. In the present series of experiments, we demonstrated that co-treatment with RASi does not only reverse Tac-induced vascular dysfunction but also abrogates the ensuing glucose metabolism disorder. Notable new findings of this study include the following: (i) RASi relieved Tac-induced glucose metabolism disorder; (ii) RASi mitigated Tac-induced β-cell dysfunction by promoting the imbalance of β-cell proliferation and apoptosis; (iii) RASi alleviated Tac-induced vascular dysfunction in dorsal pancreatic arteries and pancreatic hypoxia; (iv) Tac activated RAS by promoting renin expression and secretion, which is in turn caused by vascular dysfunction in renal arteries and the ensuing kidney hypoperfusion and hypoxia. Overall, our results identify vascular dysfunction in dorsal pancreatic arteries as a key inducer of Tac-induced glucose metabolism disorder.

The diabetogenic effects of Tac may be attributed to insufficient insulin secretion, insulin resistance or both (Triñanes et al. 2017; Ko et al. 2022; Park et al. 2023; Larsen et al. 2006). Notably, Tac itself has been show to increase insulin resitance (Larsen et al. 2006; Li et al. 2024, 2015; Sun et al. 2023; Shivaswamy et al. 2014; Ling et al. 2020; Richey et al. 1999), however, the mechanism behind it is still controversial due to different durations of Tac treatment. For example, a short-term Tac treatment (2 weeks) has been show to cause insulin resistance by downregulating GLP-1 expression in the ileum (Li et al. 2024), upregulating monocyte chemoattractant protein (MCP)−1 expression and endoplasmic reticulum stress in the adipose tissue (Sun et al. 2023), and SGLT1 expression in the jejunum (Li et al. 2015). Shivaswamy et al. also demonstrated that 2-weeks of Tac treatment leads to insulin resistance by decreasing the phosphorylation of Akt in liver and muscle (Shivaswamy et al. 2014). On the other hand, a long-term Tac treatment (8 weeks) has recently been shown to inhibit CREB-regulated transcription coactivator 2 (CRTC2) signaling that impacts hepatic metabolic homeostasis ultimately causing insulin resistance (Ling et al. 2020). In the present study, a long-term (6 months) mouse model was used to investigate mechanisms of Tac-induced diabetes. Our results show that Tac activates systemic RAS and blockage of RAS by Val alleviates Tac-induced insufficient insulin and insulin resistance. Therefore, activation of RAS plays a crucial role in long-term Tac treatment induced insulin resistance. In line with our results, previous studies have shown that Ang II infusion induces insulin resistance in dogs (Rao 1994) and rats (Rao 1994). In addition, Ang II also causes insulin resistance in cultured adipocytes by activating protein kinase C (PKC) (Gutierrez-Rodelo et al. 2022). Rats treated with RAS inhibitors (ACEi and ARB) for 2 weeks show decreased insulin resistance by restoring the composition of type I fibers in the rat soleus muscle (Higashiura et al. 2000). ARB administration in rats for 17 weeks has been show to increase energy expenditure causing weight loss and consequently alleviating insulin resistance (Müller-Fielitz et al. 2014). In obese Zucker rats, the downregulation of insulin receptor Ser994 phosphorylation after a 6-month long ARB treatment was shown to improve insulin resistance (Muñoz et al. 2006). Shinozaki et al. reported that 8 weeks ARB administered fructose-fed rats ameliorates insulin resistance by modulating vascular function (Shinozaki et al. 2004), which is similar to our results. Taken together, we conclude that long-term ARB treatment improves insulin resistance by rescuing vascular dysfunction. On the other hand, β-cell dysfunction with insufficient insulin secretion is another leading cause of Tac-induced glucose metabolism disorder (Rodriguez-Rodriguez et al. 2021). The detailed mechanisms of Tac-induced β-cell dysfunction have been elucidated by numerous studies (Rodriguez-Rodriguez et al. 2021). Tac is an effective immunosuppressor due to its ability to suppress T-cell activation and proliferation by binding to the FK-binding protein (FKBP) and subsequently inhibiting the calcineurin-NFAT (nuclear factor of activated T cells) pathway (Rodriguez-Rodriguez et al. 2021; Heit et al. 2006). The same pathway when inhibited in a β-cell specific manner in mice induces severe hyperglycemia with increasing age. Besides calcineurin-NFAT pathway, a number of other mechanisms are also involved in Tac-induced β-cell dysfunction, including insulin receptor-IRS1-PI3 K-Akt (Soleimanpour et al. 2010), mTOR (Rodriguez-Rodriguez et al. 2019), TGF-β (Wang et al. 1996), PDX-1 (Triñanes et al. 2017), MafA (Triñanes et al. 2017), NeuroD (Triñanes et al. 2017), FoxO1 (Triñanes et al. 2020) and intracellular calcium (Triñanes et al. 2017; Santulli et al. 2015). Recently, Tac-induced alterations of the gut microbiota and its metabolites and their effect on β-cell dysfunction have also been studied (Li et al. 2024; Jiao et al. 2020b). Furthermore, since pancreatic blood flow is closely linked to β-cell function, the contribution of blood flow disturbances to their dysfunction cannot be ruled out (Jansson et al. 2016; Richards et al. 2010; Obata et al. 2019; Jansson 1994). Abnormal vascular tone is a well-established occurrence in animals treated with Tac (Hoorn et al. 2011; Wang et al. 2022; Chiasson et al. 2011). Since vascular tone is an important regulator of blood flow, we proposed and tested the hypothesis that treating mice with Tac causes vascular dysfunction in their dorsal pancreatic arteries leading to low pancreatic blood flow and hence β-cell dysfunction. Our results demonstrate that this is indeed the case and underline the role of pancreatic vascular dysfunction in Tac-induced glucose metabolism disorder.

As one of the endocrine cells of the islets, β-cells produce and secrete insulin in an energy-intensive process requiring high amounts of ATP. Since ATP synthesis through mitochondrial respiration is an oxygen-intensive process, β-cells are especially sensitive to hypoxic stress leading to dysfunction and apoptosis (Jitrapakdee et al. 2010; Bensellam et al. 2016). This in combination with hyperglycemia, which itself increases oxygen demand (Ilegems et al. 2022; Sato et al. 2011; Bensellam et al. 2012), makes diabetes one of the leading causes of islet hypoxia. Furthermore, animal models of type 2 diabetes also show a decreased blood flow to the islets (Leung 2007a). Dorsal pancreatic arteries mediate the islet blood flow and thus the oxygen supply to the islets (Muratore et al. 2021). Therefore, we assumed that impaired vascular function in dorsal pancreatic arteries causes hypoxia in islets leading to β-cell dysfunction. In line with this assumption, we observed an enhanced Ang II, PE and U46619-induced contraction and impaired ACh-induced dilation of dorsal pancreatic arteries in Tac-treated mice. Meanwhile, islets isolated from Tac-treated mice showed severe cellular hypoxia, a phenomenon previously described in the kidney (Nady et al. 2024). These observations demonstrate that Tac-induced vascular dysfunction in dorsal pancreatic arteries reduces blood flow to the islet causing hypoxia, which may be an important cause of β-cell dysfunction.

There is compelling evidence that RAS is inappropriately activated by high glucose in diabetes (Luther and Brown 2011). Hyperactivated RAS plays a vital role in the development and progression of diabetic kidney disease (DKD) making RAS inhibitors (RASis) the cornerstone of DKD therapy (Luther and Brown 2011; Roscioni et al. 2014). The beneficial therapeutic effects of RASi on DKD, while independent of its antihypertensive properties (Nobakht et al. 2011), depend on its vasodilatory effect in efferent arterioles (Roscioni et al. 2014; Naaman and Bakris 2023). Besides this, RASi also has a protective effect against type 2 diabetes in high-risk populations (Luther and Brown 2011; Scheen 2004; McMurray et al. 2010). Ang II, the major biologically active component of RAS, decreases islet blood flow through its vasoconstrictive action thereby reducing insulin secretion (Luther and Brown 2011; Carlsson et al. 1998; Leung and Pancreatic 2010). Numerous basic and clinical studies have confirmed that RASi improves glucose homeostasis by preserving β-cell function and augmenting insulin secretion mainly by improving islet blood flow (Leung 2007a; Luther and Brown 2011; Kamper et al. 2010; Shree et al. 2019; Zijl et al. 2011; Kampf et al. 2005). Several mechanisms may explain the beneficial effect of RASi in maintaining glucose homeostasis. For example, RASi reverses β-cell dysfunction by regulating ABCA1 (Lyu et al. 2018), NF-κb (Chen et al. 2018), apoptosis (Tikellis et al. 2004), profibrotic (Tikellis et al. 2004) and oxidative stress (Whaley-Connell et al. 2013) signaling pathways. Since NF-κb (Sies et al. 2024), apoptosis (Sies et al. 2024) and profibrotic (Gonzalez-Gonzalez et al. 2017) signaling pathways are strongly correlated with oxidative stress, we reasoned that oxidative stress is a key mechanism for Tac-induced glucose metabolism disorder. It is well established that oxidative stress can induce apoptotic cell death (Sies et al. 2024). Therefore, RASi mitigates Tac-induced oxidative stress and the consequent overexpression of proapoptotic genes. In addition to improving β cell dysfunction, numerous studies also revealed that RASi promotes insulin release by increasing pancreatic blood flow and islet blood flow (Kampf et al. 2005; Huang et al. 2006; Olverling et al. 2013; Leung 2007b). However, the precise mechanism by which RASi enhances pancreatic blood flow and islet blood flow remains unclear. We show here that Val rescues Tac-induced decrease in pancreatic blood flow by alleviating vascular dysfunction in dorsal pancreatic arteries. Our vascular function test results are in general agreement with the previous basic and clinical studies (Lassila et al. 2000; Munkhjargal et al. 2024; Chen et al. 2024; Du et al. 2022; Tzemos et al. 2009). Oxidative stress also plays a central role in Ang II (Ajoolabady et al. 2024) and Tac-induced vascular dysfunction (Wang et al. 2022; Toral et al. 2018). Therefore, RASi mitigates Tac-induced vascular dysfunction in dorsal pancreatic arteries may be by reducing oxidative stress. In the present study, we also found that RAS was activated and Ang II was upregulated in Tac-treated mice. Taken together, it is reasonable to conclude that rescuing vascular dysfunction in dorsal pancreatic arteries using the RASi, Val, could prevent Tac-induced β-cell dysfunction and glucose metabolism disorder.

In order to gain more insight into the mechanism of Tac-induced β-cell dysfunction and glucose metabolism disorder, we next explored how Tac activates RAS. It is well established that renin, which cleaves angiotensinogen to angiotensin II, is the rate-limiting enzyme in Ang II generation (Forrester et al. 2018). The major source of systemic renin is produced and secreted from the juxtaglomerular cells of the kidney (Sequeira-Lopez and Gomez 2021). Since renal hypoxia stimulates juxtaglomerular cells to produce renin (Gomez and Sequeira-Lopez 2018; Kong et al. 2023; Krämer et al. 1998; Rathi et al. 2024), activation of RAS by Tac is possibly caused by vascular dysfunction in renal arteries and the consequent impairment of renal blood flow. This assumption was substantiated by our in vivo and in vitro experiments, we thus concluded that Tac-induced RAS activation is due to an impairment of vascular function and renal blood flow.

Although our data strongly support that inhibition of RAS can mitigate Tac-induced β-cell dysfunction and glucose metabolism disorder via improving vascular dysfunction in dorsal pancreatic arteries, we have not yet demonstrated whether RAS inhibition directly affects β-cell dysfunction induced by Tac. Another limitation of this study is the absence of clinical validation. Hence, further clinical studies will be needed to clarify this.

In conclusion, our data uncover the mechanism by which RAS inhibition protects against Tac-induced glucose metabolism disorder through alleviating: (i) the imbalance of β-cell proliferation and apoptosis; (ii) islet hypoxia; (iii) vascular dysfunction in the dorsal pancreatic arteries; (iv) low pancreatic blood flow. Therefore, RAS inhibition could be a novel therapeutic strategy for Tac-induced glucose metabolism disorder. Furthermore, this study may pave the way for new therapeutic approaches to PTDM, focusing on improving the vascular function of the dorsal pancreatic artery.

### Data sharing statement

The authors declare that all supporting data are available within the article and its online Supplemental Materials. For inquiries regarding data access or requests for additional information, interested researchers are invited to contact the corresponding author at jiangsh59@mail.sysu.edu.cn.

## Supplementary Information

Supplementary Material 1.

## Data Availability

No datasets were generated or analysed during the current study.

## Abbreviations

ACh: Acetylcholine
Ang II: Angiotensin II
CNIs: Calcineurin inhibitors
CsA: Cyclosporine A
DKD: Diabetic kidney diseases
H&E: Hematoxylin & eosin
GTT: Glucose tolerance test
HIF-1α: Hypoxia-inducible factor-1α
ITT: Insulin tolerance test
K-PSS: High potassium physiological salt solution
PE: Phenylephrine
PTDM: Post-transplant diabetes mellitus
qPCR: Quantitative polymerase chain reaction
RAS: Renin-angiotensin system
RASi: RAS inhibitor
SOT: Solid organ transplantation
Tac: Tacrolimus
T2DM: Type 2 diabetes mellitus
Val: Valsartan
