# Supplementary material for: Rescuing vascular dysfunction in dorsal pancreatic arteries prevents tacrolimus-induced glucose metabolism disorder in mice
Source: Mol Med. 2025 Jun 11;31:230. doi: 10.1186/s10020-025-01282-7 (PMC12153204; doi:10.1186/s10020-025-01282-7)
Supplement: Supplementary file 1 — Supplementary Material 1. [file 10020_2025_1282_MOESM1_ESM.docx]

# Supplementary Materials

**Rescuing vascular dysfunction in dorsal pancreatic arteries prevents tacrolimus-induced** **glucose metabolism disorder in mice**

**Short title:** Vascular dysfunction in Tac-induced hyperglycemia

Lingyan Fei^1,*^, Honghong Wang^2,*^, Dongliang Zhao^3,*^, Xiaohua Wang^1^, Jizhen Ren^4^, Lanyun Liu^4^, Chun Tang^1^, Yan Lei^1^, Qingqing Wang^5^, Yuanpeng Nie^1^, Yang Liu^1^, Na Li^1^, Ming Zhong^1^, Nan Xu^6^, Jin Wei^7^, Pontus B. Persson^8^, Andraes Patzak^8^, Pratik H. Khedkar^8,#^, Zhihua Zheng^1,#^, Shan Jiang^1,5#^

1. Department of Nephrology, Center of Kidney and Urology, the Seventh Affiliated Hospital, Sun Yat-sen University, Shenzhen 518107, China
2. Department of Physiology, School of Basic Medical Sciences, Zhejiang University School of Medicine, Hangzhou 310058, China
3. Department of Pathology, The First Affiliated Hospital of Sun Yat-Sen University, Guangzhou 510080, China
4. School of Medicine, The Sun Yat-sen University, Shenzhen, China
5. Scientific Research Center, The Seventh Affiliated Hospital of Sun Yat-Sen University, Shenzhen 518107, China
6. Department of Physiology and Pathophysiology of School of Basic Medical Sciences and Department of Cardiology of Huaihe Hospital, Henan University, Kaifeng, Henan 475004, PR China
7. Division of Nephrology at Boston Medical Center, Department of Medicine, Boston University Chobanian and Avedisian School of Medicine, Boston, MA 02118, USA
8. Institute of Translational Physiology, Charité – Universitätsmedizin Berlin, Corporate Member of Freie Universität Berlin and Humboldt-Universität zu Berlin, Charitéplatz 1, 10117 Berlin, Germany

*Lingyan Fei, Honghong Wang and Dongliang Zhao are contributed equally to this work.

^#^Corresponding authors to Pratik H. Khedkar, Institute of Translational Physiology, Charité–Universitätsmedizin Berlin, Corporate Member of Freie Universität Berlin and Humboldt-Universität zu Berlin, Charitéplatz 1, 10117 Berlin, Germany, Email: pratik.khedkar@charite.de, Zhihua Zheng, Department of Nephrology, Center of Kidney and Urology, the Seventh Affiliated Hospital, Sun Yat-sen University, Shenzhen, China, Email: [zhzhihua@mail.sysu.edu.cn](mailto:zhzhihua@mail.sysu.edu.cn), Shan Jiang, Department of Nephrology, Center of Kidney and Urology, the Seventh Affiliated Hospital, Sun Yat-sen University, Shenzhen, China, Email: jiangsh59@mail.sysu.edu.cn.

**Primer sequences**

1. Mouse Ccna2
   Forward: TCCTTGCTTTTGACTTGGCT
   Reverse: ATGACTCAGGCCAGCTCTGT
2. Mouse Hif-1α

Forward: AGGAGCCTGATGCTCTCACTCT

Reverse: TGTGTCATCGCTGCCAAAAT

1. Mouse Adm

Forward: GCAATGCTTGTTGTCCAGCC
Reverse: ACACACACACACACACACGGAAC

1. Mouse Eno1

Forward: GATGGACGGCACAGAGAATAAATC
Reverse: AGGCAGGATGACTTCAGGGTTG

1. Mouse Tpi1
   Forward: CCTTCCATTGGTTTGGGCTG
   Reverse: AATACAGGGGCTTTGGCACC
2. Mouse Hmox1
   Forward: CCACACAGCACTATGTAAAGCGTC
   Reverse: GTTCGGGAAGGTAAAAAAAGCC
3. Mouse Vegfa
   Forward: GTACCTCCACCATGCCAAGT
   Reverse: GCATTCACATCTGCTGTGCT
4. Mouse Ins1

Forward: CCCTTAGTGACCAGCTATAATCAGAGA

Reverse: ACCACAAAGATGCTGTTTGACAA

1. Mouse Ins2

Forward: CTGCTGGCCCTGCTCTTC
Reverse: AACCACAAAGGTGCTGCTTGA

1. Mouse Mafa

Forward: CCAGCTGGTATCCATGTCC
Reverse: TTCTGTTTCAGTCGGATGACC

1. Mouse Pdx1
   Forward: CGGCTGAGCAAGCTAAGGTT
   Reverse: TGGAAGAAGCGCTCTCTTTGA
2. Mouse Bcl-2
   Forward: CGCTGCGGTGCTCTTGA

Reverse: TCACACTCCGGCTTCACTGA

1. Mouse Bax
   Forward: GGAAGGCCTCCTCTCCTACTTC
   Reverse: TGAGGACTCCAGCCACAAAGA
2. Mouse Caspase3

Forward: CTGGACTGTGGCATTGAGACA

Reverse: CAGCCTCCACCGGTATCTTC

1. Mouse Ccnb1

Forward: GCGTGTGCCTGTGACAGTTA
Reverse: CCTAGCGTTTTTGCTTCCCTT

1. Mouse Ccnb2

Forward: AGCTCCCAAGGATCGTCCTC
Reverse: TGTCCTCGTTATCTATGTCCTCG

1. Mouse Ccnd1
   Forward: GCGTACCCTGACACCAATCTC
   Reverse: CTCCTCTTCGCACTTCTGCTC
2. Mouse Ccnd2

Forward: GAGTGGGAACTGGTAGTGTTG

Reverse: CGCACAGAGCGATGAAGGT

1. Mouse GAPDH

Forward: GGCCCCTCTGGAAAGCTGTGGTGT
Reverse: GTTGGGGGCCGAGTTGGGATAGG


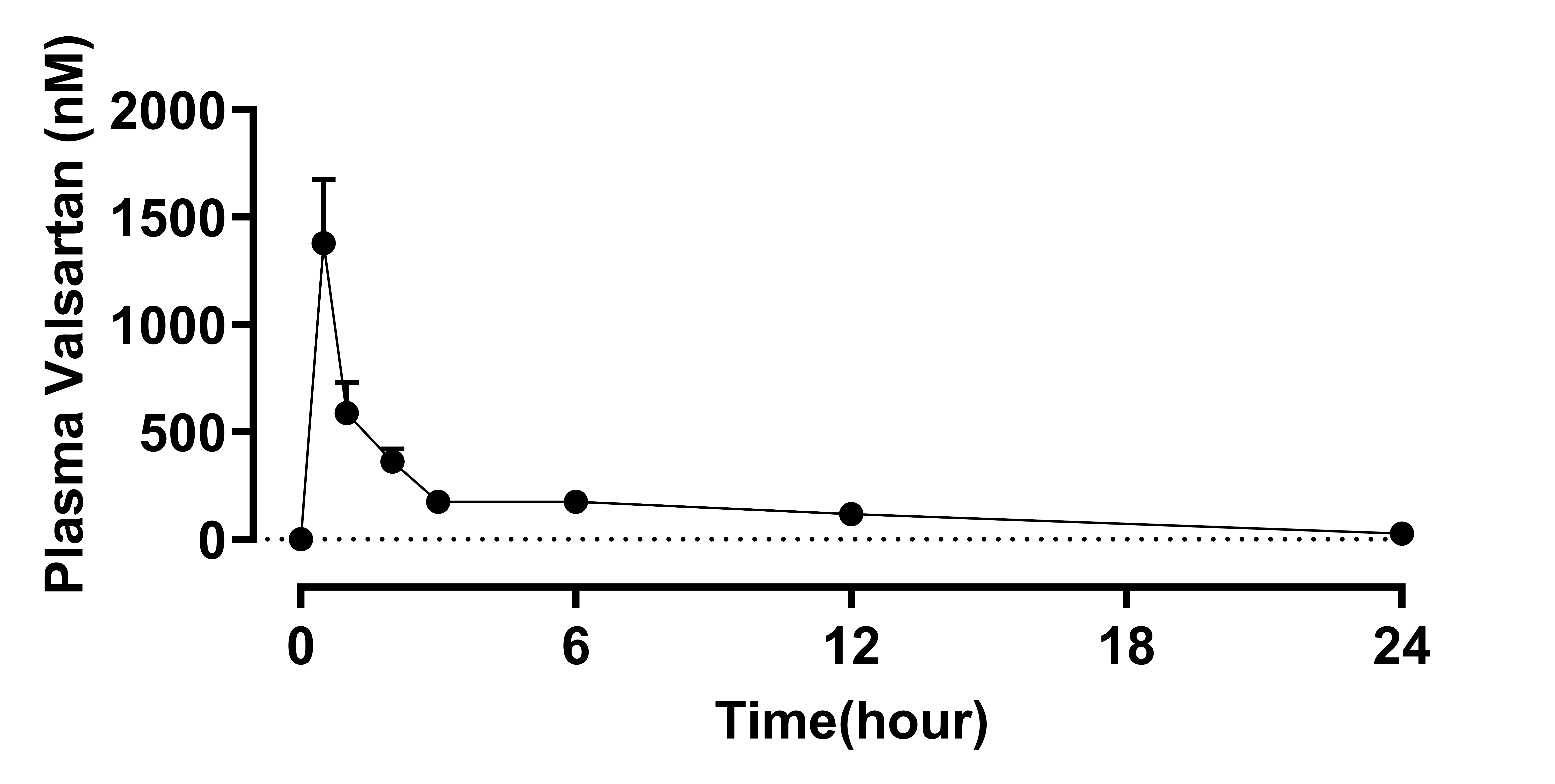


**Figure S1** The results showed that the highest concentrations of plasma valsartan (about 1.5μM) were found at 0.5 hours after valsartan gavage.


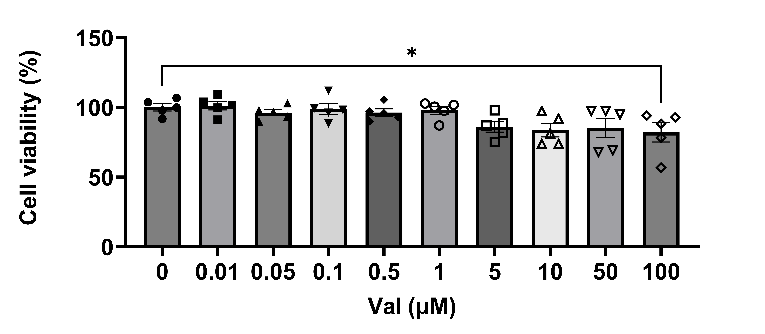


**Figure S2** HUVECs were treated with concentration gradient of valsartan for 24 hours and processed for CCK-8 assay.


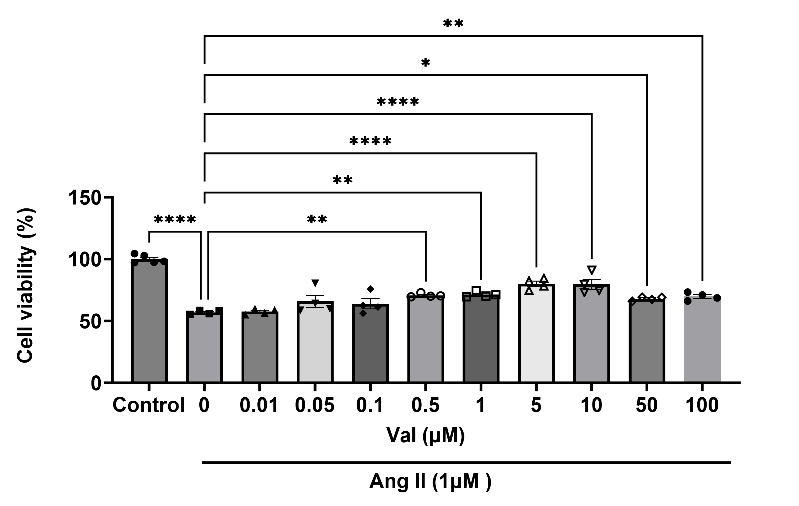


**Figure S3** HUVECs were treated with concentration gradient of valsartan with angiotensin II (Ang II) for 24 hours and processed for CCK-8 assay.


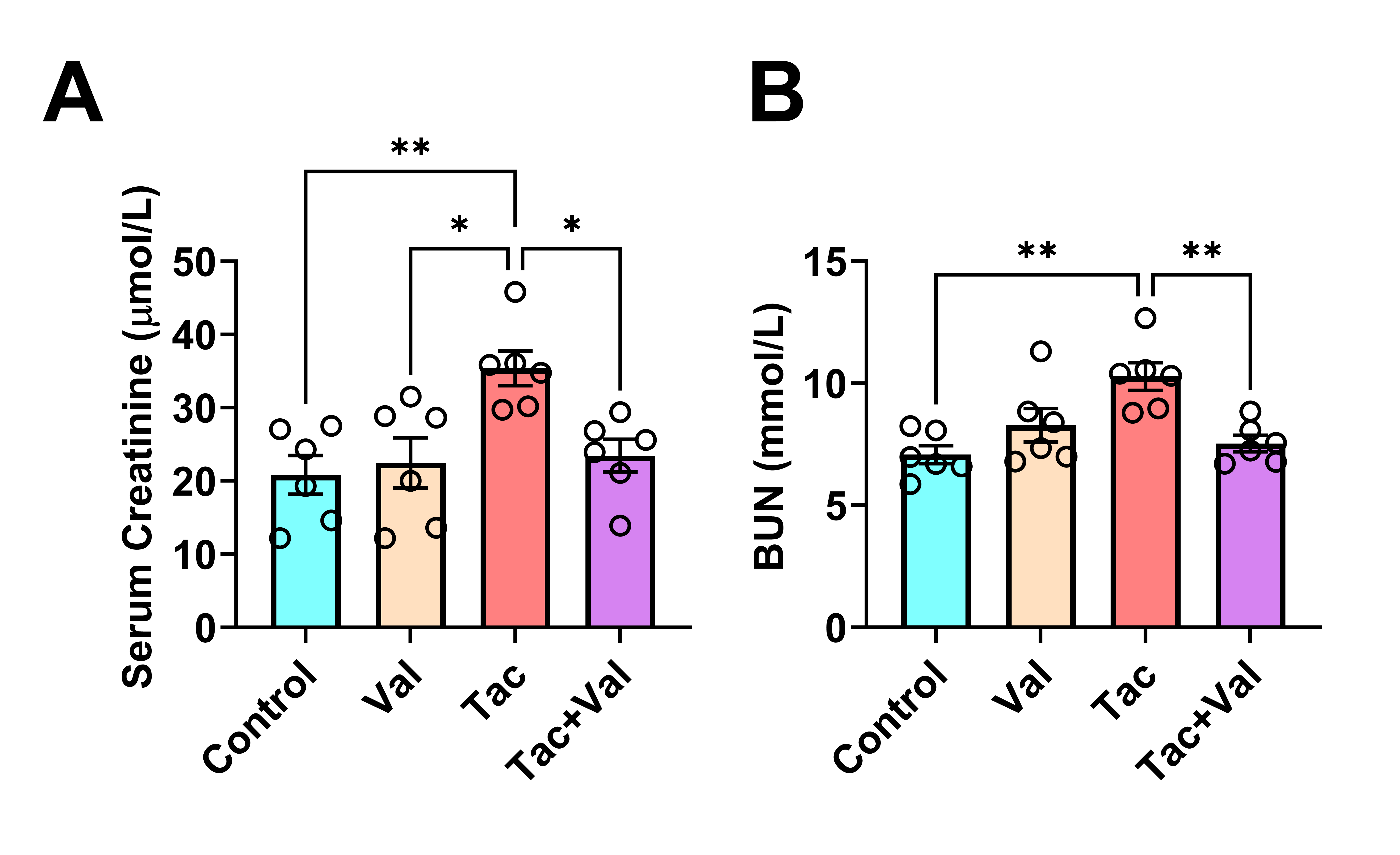


**Figure S4** The changes in serum Creatinine (A) and BUN (B) levels in mice treated with vehicle, Tac and/or Val.


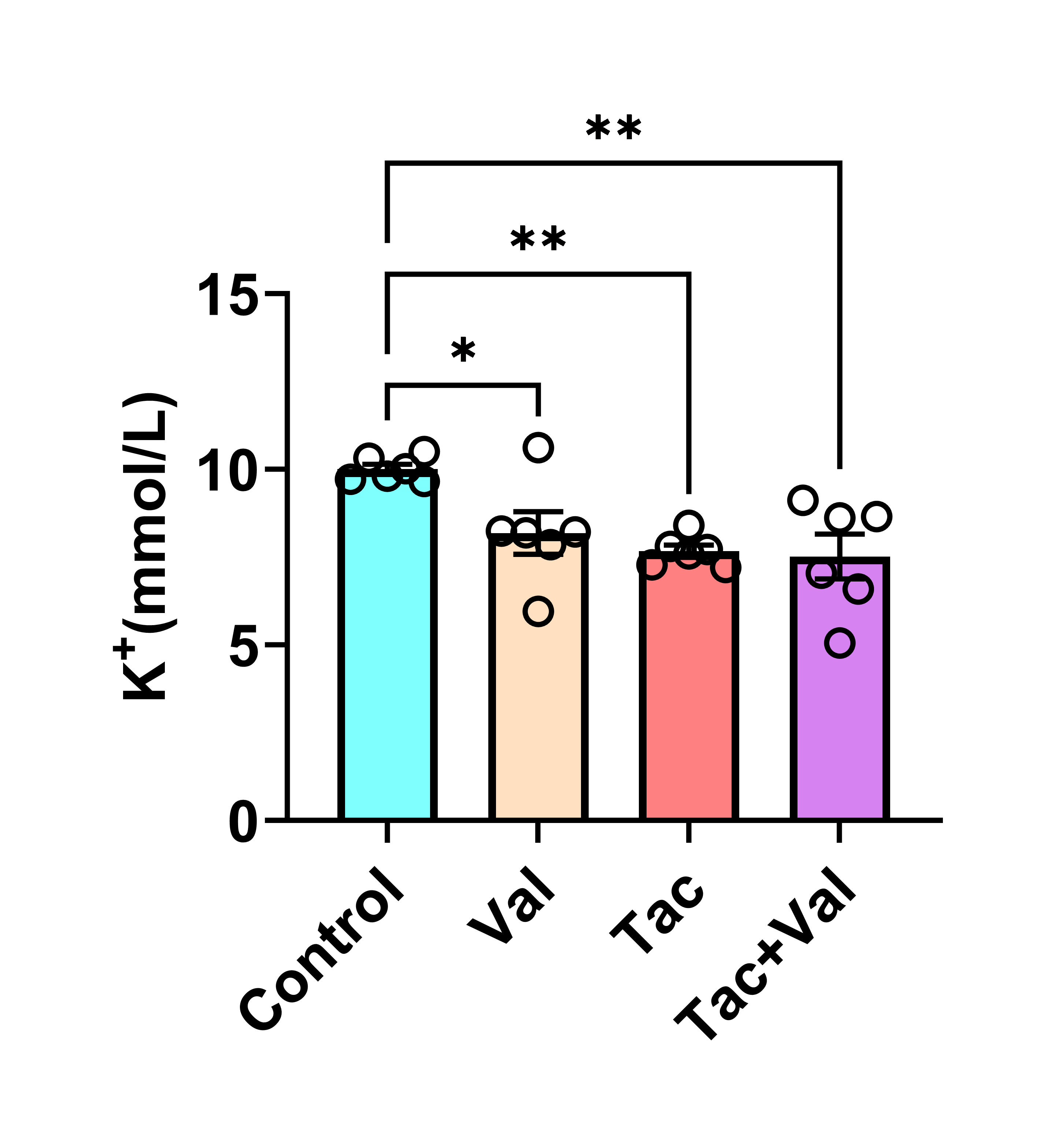


**Figure S5** The changes in serum K^+^ levels in mice treated with vehicle, Tac and/or Val.


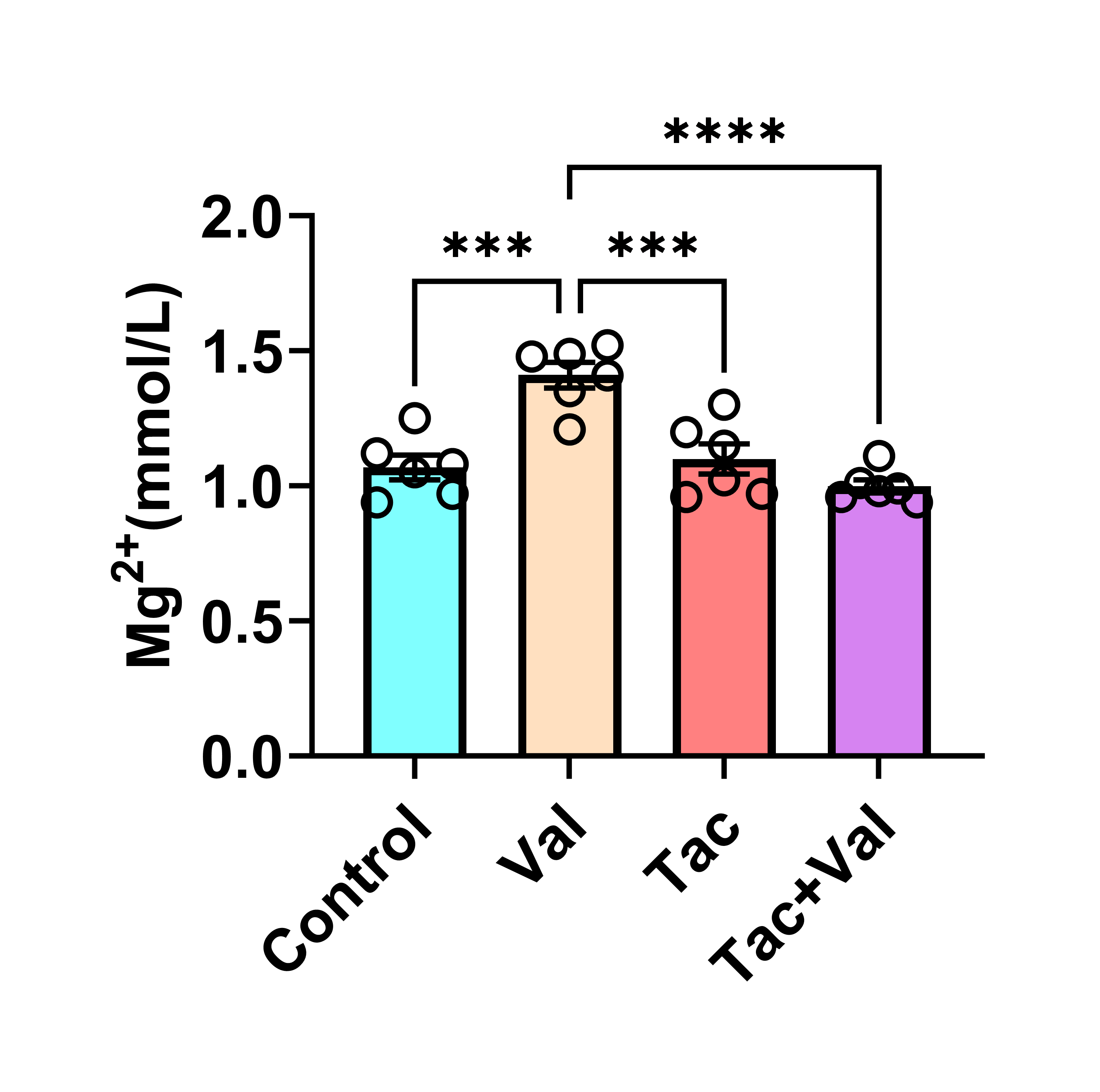


**Figure S6** The changes in serum Mg^2+^ levels in mice treated with vehicle, Tac and/or Val.
